# RETRACTION: Meta‐Analysis of the Impact of Laser Interstitial Hyperthermia on Wound Healing Complications in Brain Tumors

**DOI:** 10.1111/iwj.70488

**Published:** 2025-04-02

## Abstract

**RETRACTION:** YuP. and YangY. , “Meta‐Analysis of the Impact of Laser Interstitial Hyperthermia on Wound Healing Complications in Brain Tumors,” International Wound Journal 21, no. 1 (2024): e14628, 10.1111/iwj.14628.38272817 PMC10789519

The above article, published online on 15 January 2024, in Wiley Online Library (http://onlinelibrary.wiley.com/), has been retracted by agreement between the journal Editor in Chief, Professor Keith Harding; and John Wiley & Sons Ltd. Following an investigation by the publisher, all parties have concluded that this article was accepted solely on the basis of a compromised peer review process. The editors have therefore decided to retract the article. The authors did not respond to our notice regarding the retraction.

**RETRACTION:** P. Yu and Y. Yang , “Meta‐Analysis of the Impact of Laser Interstitial Hyperthermia on Wound Healing Complications in Brain Tumors,” International Wound Journal 21, no. 1 (2024): e14628, 10.1111/iwj.14628.38272817 PMC10789519

The above article, published online on 15 January 2024, in Wiley Online Library (http://onlinelibrary.wiley.com/), has been retracted by agreement between the journal Editor in Chief, Professor Keith Harding; and John Wiley & Sons Ltd. Following an investigation by the publisher, all parties have concluded that this article was accepted solely on the basis of a compromised peer review process. The editors have therefore decided to retract the article. The authors did not respond to our notice regarding the retraction.

